# N-[^11^C]Methyl-AMD3465 PET as a Tool for In Vivo Measurement of Chemokine Receptor 4 (CXCR4) Occupancy by Therapeutic Drugs

**DOI:** 10.1007/s11307-016-1028-8

**Published:** 2016-11-28

**Authors:** S. V. Hartimath, M. A. Khayum, A. van Waarde, R. A. J. O. Dierckx, E. F. J. de Vries

**Affiliations:** Department of Nuclear Medicine and Molecular Imaging, University of Groningen, University Medical Center Groningen, Hanzeplein 1, P.O. Box 31.001, 9713 GZ Groningen, The Netherlands

**Keywords:** Pet, Logan graphical analysis, Compartment modeling, Receptor occupancy, CXCR4 receptors imaging

## Abstract

**Purpose:**

Chemokine receptor 4 (CXCR4) is overexpressed in many cancers and a potential drug target. We have recently developed the tracer N-[^11^C]methyl-AMD3465 for imaging of CXCR4 expression by positron emission tomography (PET). We investigated the pharmacokinetics of N-[^11^C]methyl-AMD3465 in rats bearing a C6 tumor and assessed whether the CXCR4 occupancy by the drug Plerixafor® can be measured with this PET tracer.

**Procedure:**

A subcutaneous C6 tumor was grown in Wistar rats. Dynamic N-[^11^C]methyl-AMD3465 PET scans with arterial blood sampling was performed in control rats and rats pretreated with Plerixafor® (30 mg/kg, s.c). The distribution volume (*V*_T_) of the tracer was estimated by compartment modeling with a two-tissue reversible compartment model (2TRCM) and by Logan graphical analysis. The non-displaceable binding potential (BP_ND_) was estimated with the 2TRCM. Next, CXCR4 receptor occupancy of different doses of the drug Plerixafor® (0.5–60 mg/kg) was investigated.

**Results:**

The tumor could be clearly visualized by PET in control animals. Pretreatment with 30 mg/kg Plerixafor® significantly reduced tumor uptake (SUV 0.65 ± 0.08 vs. 0.20 ± 0.01, *p* < 0.05). N-[^11^C]Methyl-AMD3465 was slowly metabolized in vivo, with 70 ± 7% of the tracer in plasma still being intact after 60 min. The tracer showed reversible in vivo binding to its receptor. Both 2TRCM modeling and Logan graphical analysis could be used to estimate *V*_T_. Pre-treatment with 30 mg/kg Plerixafor® resulted in a significant reduction in *V*_T_ (2TCRM 0.87 ± 0.10 vs. 0.23 ± 0.12, *p* < 0.05) and BP_ND_ (1.85 ± 0.14 vs. 0.87 ± 0.12, *p* < 0.01). Receptor occupancy by Plerixafor® was dose-dependent with an in vivo ED_50_ of 12.7 ± 4.0 mg/kg. Logan analysis gave comparable results.

**Conclusion:**

N-[^11^C]Methyl-AMD3465 PET can be used to visualize CXCR4 expression and to calculate receptor occupancy. *V*_T_ determined by Logan graphical analysis is a suitable parameter to assess CXCR4 receptor occupancy. This approach can easily be translated to humans and used for early drug development and optimization of drug dosing schedules.

## Introduction

Chemokine receptor 4 (CXCR4) is a member of the α-chemokine receptor family, which is involved in maintaining body homeostasis, hematopoiesis, immune cell trafficking, and homing and retention of stem cells [1, 2]. However, CXCR4 and its natural ligand stromal derived factor-1α (CXCL12) also play a crucial role in the development, proliferation, and metastasis of cancer [3]. Elevated expression of CXCR4 has been observed in more than 20 different human tumor types [4–6], and the level of CXCR4 expression was associated with tumor grade and poor prognosis [7, 8]. CXCR4 has been considered as a target for (adjuvant) cancer therapy [9, 10]. Inhibition of CXCR4-mediated signaling has therapeutic efficacy by itself, but can also have a synergistic effect with other anti-cancer drugs, such as bortezomib, melphalan, doxorubicin, and dexamethasone [11].

In drug development, it is essential to know the kinetics and the in vivo binding properties of a drug to its target at an early stage, preferably in animal studies or in early clinical trials. Usually, there is a threshold for the percentage of the receptors that have to be occupied by the drug in order to achieve a pharmacological effect. Currently, drug-dosing regimens for patients are mainly based on the outcome of preclinical and phase I-II studies. However, these studies cannot elucidate the relationship between amount of drug administered and the occupancy of the receptors in vivo.

Positron emission tomography (PET) can be used to noninvasively measure receptor occupancy in vivo by comparing the uptake of a specific tracer in the tissue of interest before and after administration of one or more doses of the drug under investigation. These studies can answer many critical questions, such as whether the drug has reached its target, the level of receptor occupancy after administration of a therapeutic dose, and the duration of receptor binding by the drug. With this information, effective dosing regimens can be designed for the expensive phase 3 clinical trials.

Recently, we have developed N-[^11^C]methyl-AMD3465 as a radiotracer for PET imaging of CXCR4 receptors [12]. The aim of this study is to investigate various methods for quantification of CXCR4 availability using N-[^11^C]methyl-AMD3465 PET in immune-competent tumor-bearing rats. We also investigated whether this approach can be applied to measure CXCR4 receptor occupancy of the drug Plerixafor® (AMD3100 8HCl). To date, no report has been published on the estimation of CXCR4 receptor occupancy by PET.

## Material and Methods

### General

All reagents and solvents were obtained from commercial suppliers and used without further purification. AMD3100 octahydrochloride (Plerixafor®) was prepared as previously described [13, 14]. A stock solution of Plerixafor® was prepared in phosphate buffered saline (PBS) and neutralized with 1 M NaOH. N-[^11^C]Methyl-AMD3465 was prepared as previously described [12]. C6 rat glioma cells (ATCC, Manassas, VA) were cultured in monolayers in Dulbecco’s Modified Eagle Medium, supplemented with 10% fetal calf serum. Cells were maintained in a humidified atmosphere with 5% CO_2_ at 37 °C.

### Animal Model

All animal experiments were performed in compliance with the Dutch law on animal experiments. The protocol was approved by the Institutional Animal Care and Use Committee of the University of Groningen. Male Wistar rats (*n* = 34, 300 ± 20 g, Harlan) were maintained at a 12-h day and 12-h night regime and fed standard laboratory chow ad libitum. Rat C6 glioma cells (3 million) in a mixture of Matrigel and complete medium (1:1 (*v*/*v*)) were subcutaneously injected into the right shoulder of the animal. Solid tumors (350 ± 20 mg) were allowed to grow for 5–6 days before the animals underwent PET experiments.

The study was divided into two parts: first, different pharmacokinetic modeling approaches to quantify N-[^11^C]methyl-AMD3465 uptake were evaluated, then the CXCR4 receptor occupancy of the drug Plerixafor was investigated. Body weights and injected doses are shown in Table 1.

**Table 1:** Body weight and injected dose of the tracer. Animals were treated with saline or different doses of Plerixafor 30 min before tracer injection. Data are reported as mean ± standard deviation

Group	Plerixafor® dose	Body weight	Injected dose	
Kinetic modeling study		g	MBq	nmol
Control (*n* = 6)	Saline	351 ± 31	30 ± 5	0.65 ± 0.12
Pretreated (*n* = 6)	30 mg/kg s.c	320 ± 25	36 ± 8	0.76 ± 0.20
Receptor occupancy study				
Dose-1 (*n* = 3)	0.5 mg/kg. s.c	301 ± 16	32 ± 4	0.68 ± 0.06
Dose-2 (*n* = 3)	3.0 mg/kg. s.c	326 ± 20	35 ± 5	0.74 ± 0.10
Dose-3 (*n* = 3)	10 mg/kg. s.c	317 ± 19	38 ± 8	0.80 ± 0.19
Dose-4 (*n* = 3)	30 mg/kg. s.c	315 ± 15	31 ± 2	0.69 ± 0.05
Dose-5 (*n* = 3)	60 mg/kg. s.c	335 ± 13	34 ± 4	0.72 ± 0.08

### Small Animal PET

Control animals (also used as baseline scan for occupancy studies, *n* = 6) were anesthetized with a mixture of isoflurane/air (5% induction and 2% maintenance, Teva Pharmachemie, The Netherlands). A cannula was placed in the femoral artery for rapid blood sampling, and another cannula was inserted in the femoral vein for tracer injection. Two animals were simultaneously place in the PET camera (Focus-220, Siemens-Concorde). A transmission scan with a Co-57-point source was made for attenuation and scatter correction. After the transmission scan, 30 ± 5 MBq of N-[^11^C]methyl-AMD3465 in a volume of 1 ml was injected. The injection was performed with a syringe pump set at a speed of 1 ml/min. The PET scan was started when the tracer started to enter the body of the first animal. The second animal was injected 16 min later. A 76-min list mode protocol was used for data acquisition. A series of 15 blood samples (0.10–0.15 ml) was drawn at 10, 20, 30, 40, 50, 60, 90, 120, 180, 300, 450, 600, 900, 1800, and 3600 s after tracer injection. After a blood sample was taken, 0.15 ml of heparinized saline was injected via the artery cannula to prevent hypovolemia and large changes in blood pressure. Radioactivity in 25 μl of plasma and 25 μl of whole blood were determined and used as an arterial input function for pharmacokinetic modeling.

To examine specific binding, six rats were pretreated with Plerixafor® (30 mg/kg, s.c.), 30 min before the tracer injection. Scanning was performed as described above. After the scan, the animals were terminated under deep anesthesia. List mode data of the emission scan was separated into 21 frames (6 × 10, 4 × 30, 2 × 60, 1 × 120, 1 × 180, 4 × 300, 3 × 600 s). Emission sinograms were iteratively reconstructed (OSEM2D, 16 subsets, 4 iterations) after being normalized and corrected for attenuation, scatter, and radioactive decay. The data sets consisted of 95 slices, with a slice thickness of 0.8 mm and an in-plane 128 × 128 image matrix with a pixel size of 1.1 mm. Images were smoothed with a 1 mm Gaussian filter. Preliminary volumes of interest (VOIs) were manually drawn around the tumor on the summed PET images using the drawing tool of the software. The final VOIs were generated automatically by the software using 75% of the maximum uptake in the preliminary VOI of the tumor as the threshold. For each VOI, a time activity curve (TACs) was calculated using Inveon Research Workplace software (Siemens-Concorde). Tracer uptake was normalized to the injected tracer dose and body weight and is presented as standardized uptake value (SUV). The SUV values presented in this manuscript represent the SUV_mean_ within the 75% threshold ROI.

### In Vivo Metabolite Analysis

For metabolite analysis, a group of rats (*n* = 6) were injected with 50 ± 5 MBq of tracer while being anesthetized with a mixture of isoflurane/air (5% induction and 2% maintenance). A series of 0.5–0.8 ml arterial blood samples was drawn at 5, 15, 30, 40, and 60 min after tracer injection. Blood samples were centrifuged at 13,000 rpm for 2 min. Plasma was collected, 0.1 mL of 70% H_3_PO_4_ was added, and the solution was vortexed for 30 s. The mixture was centrifuged for 2 min at 13,000 rpm, and the clear supernatant was collected. The supernatant was analyzed by HPLC (Shimadzu), using a μBondapak column [7.8 × 300 mm, Waters] and water/acetonitrile (90/10 *v*/*v*) as the eluent. The flow rate was set at 3 ml/min, and samples were collected at time intervals of 30 s. The radioactivity in the collected samples was measured with a gamma counter (Compugamma CS1282, LKB-Wallac, Turku, Finland), and the percentage of intact tracer was calculated. An average metabolite curve was generated by fitting the data points of all animals with an exponential function. Metabolite-corrected plasma curves were generated by correcting the plasma curves of the individual animals for the percentage of intact tracer, using the population-based metabolite curve. The metabolite-corrected plasma curves of individual animals were used as an input function curve for pharmacokinetic modeling.

### Compartment Modeling

Tracer kinetics of N-[^11^C]methyl-AMD3465 was analyzed by compartmental modeling using the 1-tissue compartment model (1TCM) and the 2-tissue reversible compartment model (2TRCM). The whole blood curve and the metabolite-corrected plasma curve were used as input functions; the blood volume fraction (*V*b) was not fixed. Inveon workstation software (Siemens) was used to estimate the model parameters (rate constants, *V*b). Tracer uptake was expressed as *V*_T_ and non-displaceable binding potential (BP_ND_), which were defined as [15, 16].

$$ {V}_{\mathrm{T}}=\frac{K_1}{k_2}\left(1+B{P}_{ND}\right)\kern0.5em B{P}_{ND}=\frac{k_3}{k_4} $$

### Graphical Analysis

Graphically, analysis was performed on the TACs of the tumor, using the Logan and Gjedde–Patlak graphical methods. The whole blood and the metabolite-corrected plasma curves were used as input functions to calculate the total volume of distribution (*V*_T_) in the tumor.

### Receptor Occupancy

CXCR4 occupancy was investigated in C6 tumor-bearing rats that were subcutaneously injected with different doses of Plerixafor® (0.5, 3, 10, 30, and 60 mg/kg, *n* = 3/dose). After 30 min, N-[^11^C]methyl-AMD3465 was administered and a 60-min dynamic PET scan with arterial blood sampling was performed as described above. Compartment modeling with the 2TRCM and Logan graphical analysis were used to determine the *V*_T_ in the tumor. Scans from control animals were used as baseline scans, because repetitive PET scans with arterial blood sampling in the same animal was not possible. The occupancy of the drug was calculated from the average *V*_T_ of control animals (*V*_T_ (baseline)), the *V*_T_ of animals that were administered the drug (*V*_T_ (drug)) and the non-displaceable binding (*V*_ND_). The *V*_ND_ was estimated by extrapolating the fit of the *V*_T_ versus drug dose curve to an infinite drug dose.

The occupancy of Plerixafor® was estimated according to the formula:

$$ \mathrm{Occupancy}=\left(\frac{\left[V\mathrm{t}\left(\mathrm{baseline}\right)-V\mathrm{t}\left(\mathrm{drug}\right)\right]}{V\mathrm{t}\left(\mathrm{baseline}\right)-{V}_{ND}}\right)\times 100\% $$

In addition, the drug occupancy was calculated using the BP_ND_, according to the formula:

$$ \mathrm{Occupancy}=\left(\frac{B{P}_{ND}\left(\mathrm{baseline}\right)-B{P}_{ND}\left(\mathrm{drug}\right)}{B{P}_{ND}\left(\mathrm{baseline}\right)}\right)\times 100\% $$

## Statistical Analysis

All results are expressed as mean ± standard deviation. Statistical analyses were performed using the two-sided unpaired students’*t* test in GraphPad Prism 5. A probability (p) value <0.05 was considered statistically significant.

## Results

### PET Imaging

The N-[^11^C]methyl-AMD3465 PET images (0–60 min) of a control rat with a C6 tumor and a rat pretreated with 30 mg/kg Plerixafor® are represented in Fig. 1. The C6 tumor is clearly visible in the control animal, but not in the Plerixafor® treated animal, indicating that the drug is able to compete with the PET tracer for the binding site of CXCR4. Pretreatment with 30 mg/kg Plerixafor® significantly reduced the tracer uptake in the tumor (SUV 0.65 ± 0.08 vs. 0.20 ± 0.01, *p* < 0.05). Figure 1 shows that pretreatment with the CXCR4 antagonist also reduces tracer uptake in the salivary glands, which can be explained by the relatively high expression levels of the CXCR4 receptors in these glands [17].

**Fig. 1.**
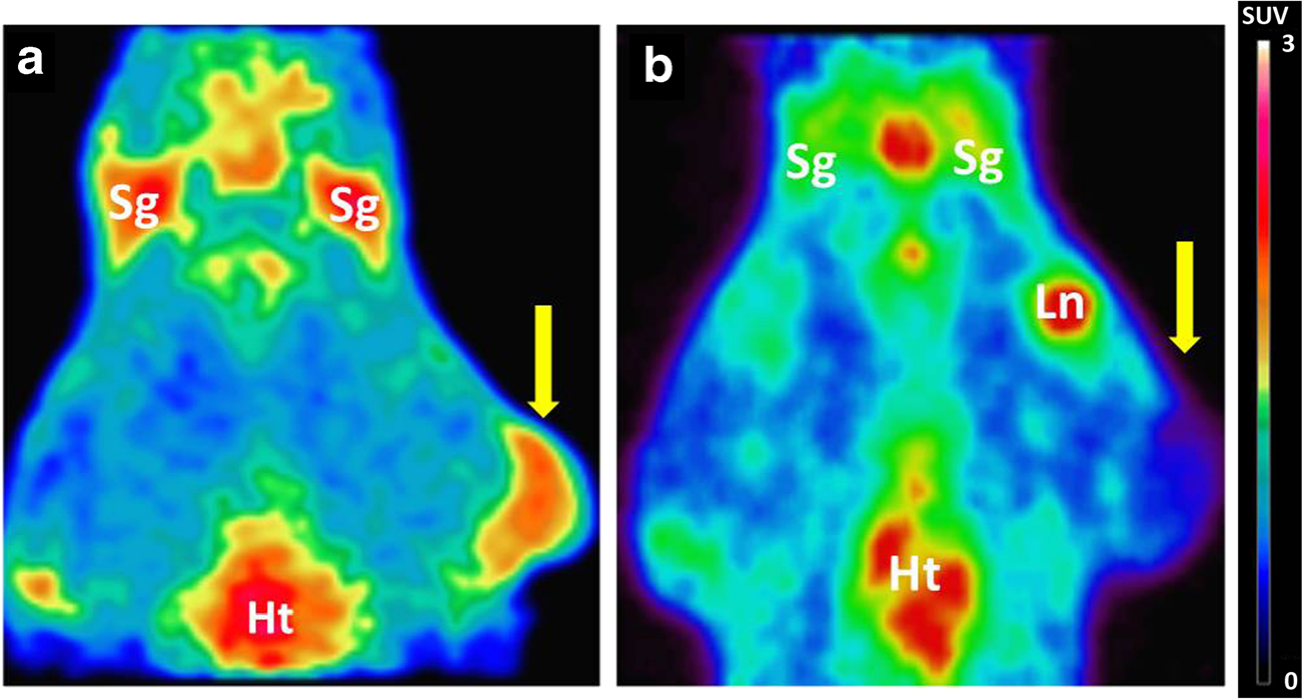
Coronal PET images (0–60 min) of C6 tumor-bearing Wistar rats.**a** Control animal (baseline) with the tumor and heart in the field of view. **b** Animal pre-treated with AMD3100 8HCl (Plerixafor® 30 mg/kg), 30 min before the tracer injection. The *arrow* indicates the position of the tumor; *Sg* Salivary gland, *Ln* Lymph node and *Ht* Heart.

### Tracer Kinetics

**Fig. 2:**
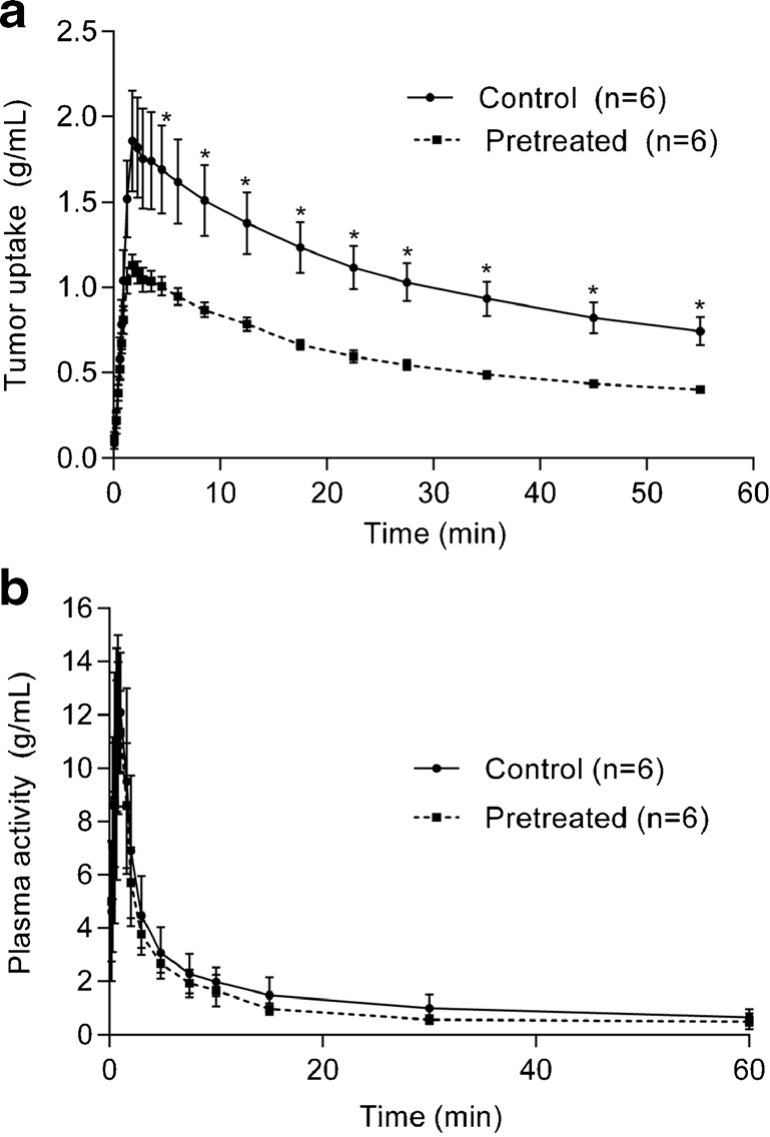
**a** Time-activity curves of the C6 tumor in Wistar rats (*n* = 6). Animals were either untreated (control) or injected with 30 mg/kg Plerixafor® 30 min before the tracer injection (pretreated). The uptake of the tracer is expressed as SUV and corrected for injected dose, body weight and decay. **b** Kinetics of N-[^11^C]methyl-AMD3465 in plasma. Plasma data were corrected for the metabolites. Pre-treatment with Plerixafor® did not significantly affect the kinetics of the tracer in plasma. Error bars represent standard deviations. Statistically significant differences are indicated by * (*p* < 0.05).

Figure 2 presents the average TACs of N-[^11^C]methyl-AMD3465 in the tumor of control and Plerixafor® pretreated animals. Tracer uptake in the tumor reached a maximum at 30 s after injection, followed by a bi-exponential clearance in both groups. The clearance half-lifes of the tracer from the tumor were 10 ± 1 min (10%) and 129 ± 9 min (90%) in control rats and 8 ± 3 min (14%) and 6 8 ± 5 min (86%) in Plerixafor®-treated animals. Pretreatment with Plerixafor® resulted in significantly (*p* < 0.05) lower activity levels in the tumor of pretreated animals than in tumors of control animals beyond 5 min after tracer injection (Fig. 2a). The clearance of the tracer from plasma was not affected by the drug treatment (Fig. 2b), as plasma activity was not significantly different between control animals and Plerixafor® pretreated rats at any time point.

### Metabolite Analysis

**Fig. 3.**
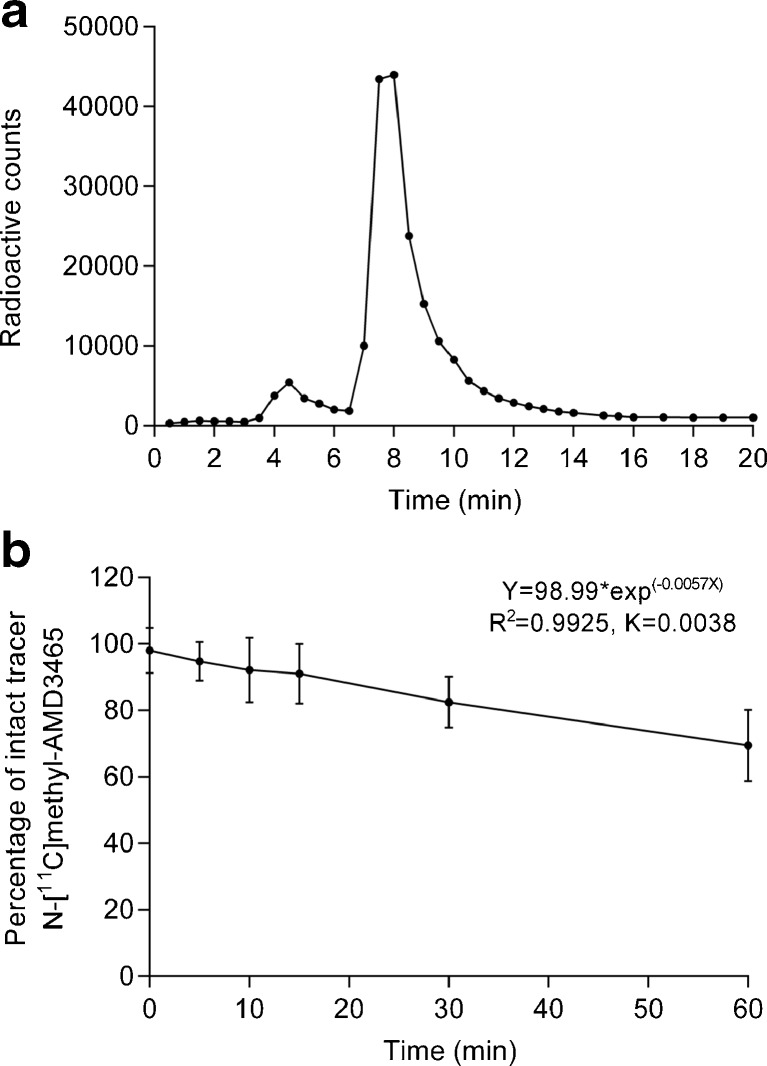
In vivo metabolite analysis (*n* = 6). **a** Chromatogram representing the plasma metabolite analysis by HPLC. **b** Graph showing the percentage of intact tracer in plasma as a function of time. Error bars represent standard deviations.

HPLC analysis of plasma samples showed the formation of one radioactive metabolite that was more hydrophilic than N-[^11^C]methyl-AMD3465 (retention times: N-[^11^C]methyl-AMD3465 8 min, radioactive metabolite 5 min; Fig. 3a). The tracer was slowly metabolized in vivo, and metabolism was highly reproducible between animals. The percentage of intact tracer decreased from 98 ± 2% at 5 min to 70 ± 7% at 60 min (Fig. 3b).

## Compartment Modeling

TACs generated from the VOI of the tumor were analyzed with two reversible compartment models: 1TCM and 2TRCM. The Akaike information criterion values (AIC) values were 423 ± 28 and 296 ± 13 for the 1TCM and 2TRCM, respectively, indicating that tracer kinetics could be better fitted with the 2TRCM. Therefore, the 2TRCM was used to estimate the parameters *K*1, *k*2, *k*3, *k*4, and *V*b, which were subsequently converted into *V*_T_ and BP_ND_ (Table 2). Animals treated with 30 mg/kg s.c. Plerixafor® showed a significantly lower BP_ND_ than control animals (1.85 ± 0.14 vs. 0.87 ± 0.12, *p* < 0.01). Similarly, the *V*_T_ was significantly reduced after pretreatment with Plerixafor® (79%, *p* < 0.05). Pretreatment with Plerixafor did not affect the curve fit, as tracer kinetics could still be fitted by the 2TRCM. Compartment modeling with the 2TRCM indicated that the estimated total blood volume (*V*_b_) was not affected by Plerixafor® treatment (0.07 ± 0.04 vs. 0.06 ± 0.03 in control and drug-treated animals, respectively).

**Table 2:** Results of kinetic modeling of N-[^11^C]methyl-AMD3465 tumor kinetics in C6 glioma-bearing Wistar rats. Animals were either untreated (control) or treated with 30 mg/kg Plerixafor® 30 min prior to tracer injection. Data are presented as a mean ± standard deviation

Parameter	Control	Plerixafor® (*n* = 6, 30 mg/kg)	Statistical significance	Method
	(*n* = 6)			
Compartment modeling				
*V*_T_	0.87 ± 0.10	0.23 ± 0.12	*p* < 0.05	2TRCM
BP	1.85 ± 0.14	0.87 ± 0.12	*p* < 0.01	
*V*_b_	0.07 ± 0.04	0.06 ± 0.03	NS	
Graphical analysis				Logan
V_T_	0.79 ± 0.16	0.18 ± 0.10	*p* < 0.05	

*NS* not significant

## Graphical Analysis

**Fig. 4.**
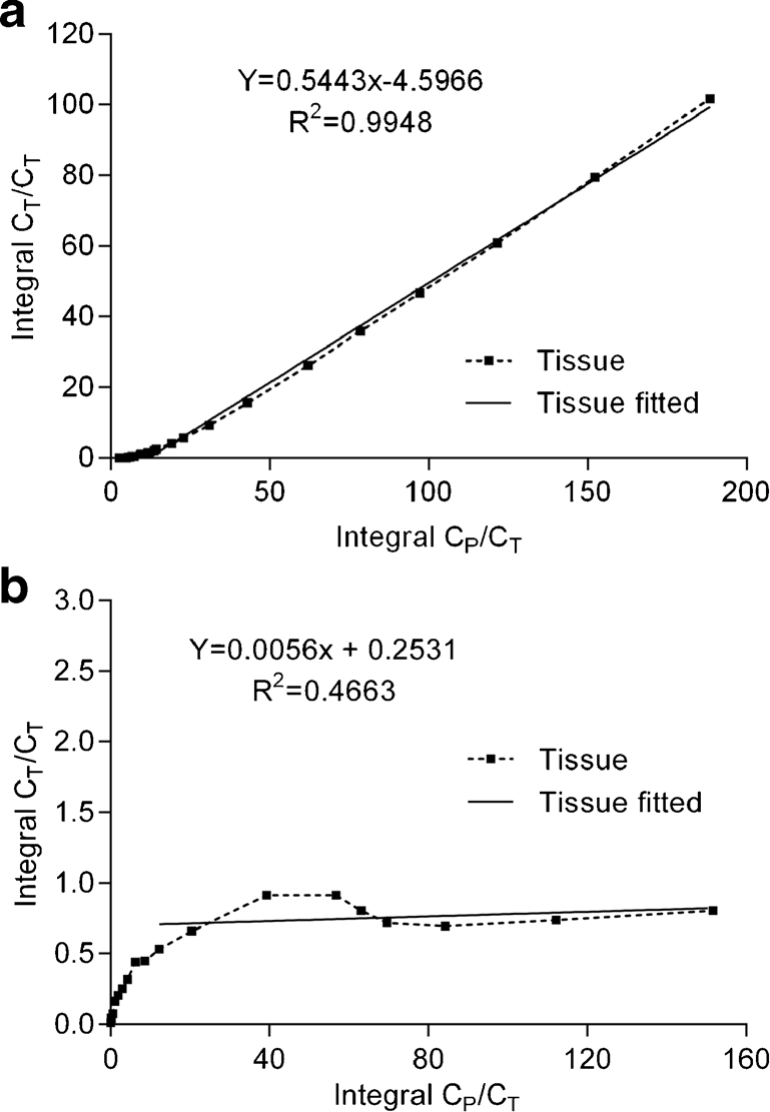
Representative graphical analyses of tracer uptake in the C6 tumor. **a** Logan graphical analysis using a 10-min delay time. **b** Patlak graphical analysis with a delay time of 20 min.

Figure 4 shows representative examples of a Logan and a Patlak plot of N-[^11^C]methyl-AMD3465 uptake in the tumor. The tracer kinetics showed a better fit with Logan graphical analysis (*R* ^2^ = 0.99 ± 0.08) than with Patlak analysis (*R* ^2^ = 0.47 ± 0.92), confirming that the binding of N-[^11^C]methyl-AMD3465 to CXCR4 receptors is reversible. The *V*_T_ calculated by Logan graphical analysis was significantly lower in rats pretreated with 30 mg/kg s.c. Plerixafor® than in control rats (0.18 ± 0.10 vs. 0.79 ± 0.16, *p* < 0.05), indicating that the tracer displays specific binding to the CXCR4 receptor (Table 2).

## Receptor Occupancy

**Fig. 5.**
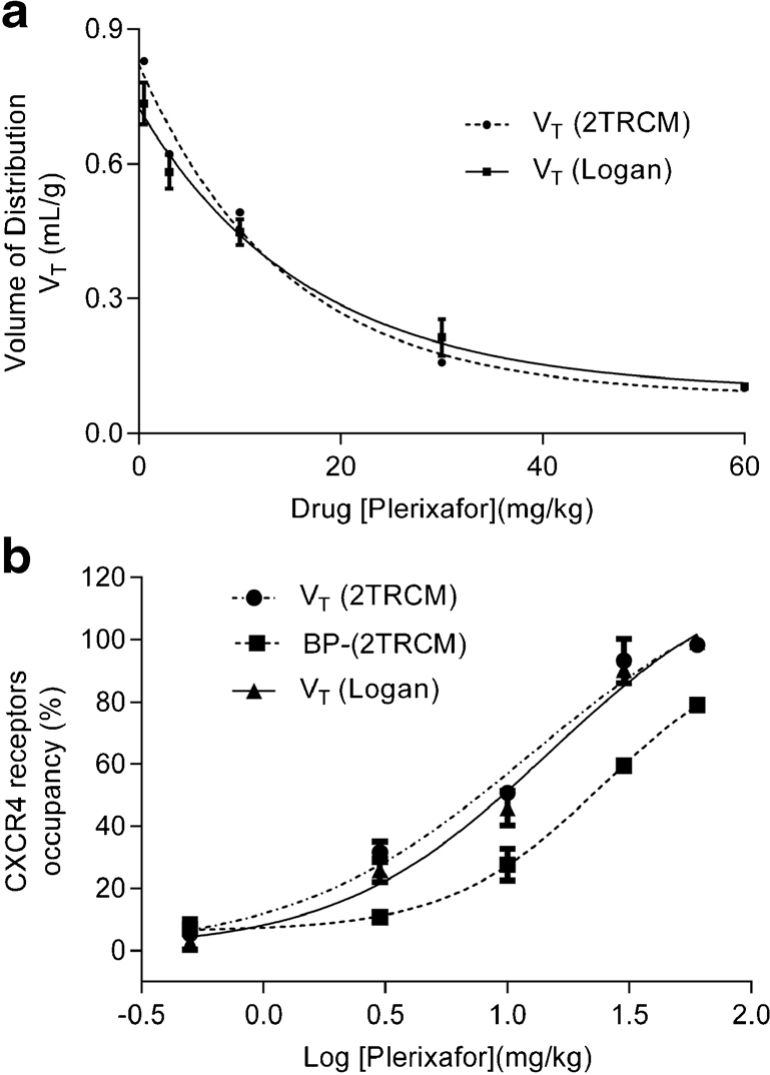
Receptor occupancy graphs. **a** The receptor occupancy was estimated by non-linear regression analysis. The tumor distribution volume (*V*_T_) derived from 2TRCM and Logan was used to calculate the nonspecific volume of distribution (*V*_ND_) by extrapolating to an infinite drug dose. **b** Estimation of the ED_50_ from the receptor occupancy.

In order to estimate drug occupancy from *V*_T_ values at different drug concentrations, first, the *V*_ND_ in tumor was calculated by plotting the *V*_T_ (dose) versus the drug concentration and extrapolating the curve to infinite drug concentration (Fig. 5a). The estimated *V*_ND_ determined from 2TRCM, and Logan derived *V*_T_ values was 0.088 ± 0.041 and 0.094 ± 0.034, respectively (Table 3). Occupancy estimates based on *V*_T_ values calculated from 2TRCM analysis and Logan analysis gave comparable results, and almost 100% occupancy was already obtained at doses >30 mg/kg. In contrast, the occupancy calculated from the BP_ND_ reached only 80% at the highest dose of 60 mg/kg (Fig. 5b, Table 3). Occupancy determined from BP_ND_ values correlated poorly with the occupancy calculated from *V*_T_ values.

**Table 3:** Percentage of receptor occupancy calculated from the volume of distribution (*V*_T_) and non-displaceable binding potential (BP_ND_) obtained from 2TRCM and Logan graphical analysis. The C6 tumor-bearing Wistar rats were treated with different concentrations of Plerixafor 30 min before tracer injection. All data are expressed as the mean ± standard deviation

Drug concentration (*n* = 3, mg/kg)	Occupancy (%) (*V*_T_-2TRCM)	Occupancy (%) (BP_ND_-2TRCM)	Occupancy (%) (V_T_-Logan)
0.5	5 ± 4	6 ± 3	2 ± 1
3	31 ± 3	10 ± 1	26 ± 4
10	50 ± 2	27 ± 5	46 ± 5
30	93 ± 7	59 ± 2	90 ± 2
60	98 ± 1	79 ± 1	99 ± 1
ED_50_ (mg/kg)	12.7 ± 4.0	23.8 ± 4.2	14.2 ± 2.3
*V*_ND_	0.088 ± 0.041	N.A.	0.094 ± 0.034

*N.A.* not applicable

A non-linear regression analysis was used to estimate the ED_50_ of Plerixafor® in the living animal. Thus, the in vivo ED_50_ of the drug were 12.7 ± 4.0, 23.8 ± 4.2 and 14.2 ± 2.3 mg/kg when the occupancy was estimated from the *V*_T_ and BP_ND_ derived from 2TRCM, and *V*_T_ derived from Logan graphical analysis, respectively. The non-linear regression of occupancy data derived from the BP_ND_ did not reach a statistically significant curve fit (*p* = 0.29.) and therefore the ED_50_ estimated by this method should be considered as unreliable.

## Discussion

In this study, we showed that occupancy of CXCR4 receptors by the drug Plerixafor® can be measured in vivo in C6 glioma-bearing rats, using PET and kinetic modeling. Plerixafor® is a potent and selective CXCR4 receptor antagonist, which was originally developed as an anti-HIV drug, but nowadays, it is also used as a drug in adjuvant cancer therapy and to stimulate the mobilization of hematopoietic stem cells from bone marrow for autologous transplantation. Using N-[^11^C]methyl-AMD3465 PET, we assessed CXCR4 receptor occupancy by the drug and calculated its ED_50_ in vivo*.*

Pretreatment with Plerixafor® (30 mg/kg) resulted in a significant reduction in the uptake of the tracer in the tumor, indicating that N-[^11^C]methyl-AMD3465 shows specific binding to CXCR4 receptors. Tracer kinetics in blood and plasma were not affected by pretreatment with Plerixafor®. We used only population-based metabolite correction (PBMC), as we only observed little variation within the population (Fig. 3b). In vivo metabolite analysis of plasma showed that the tracer was slowly metabolized in vivo*,* as approximately 70% of N-[^11^C]methyl-AMD3465 was still intact 1 h after tracer injection. Only one polar radioactive metabolite was detected in plasma (Fig. 3a). MetaPrint2D software (Cambridge) predicted that this metabolite of N-[^11^C]methyl-AMD3465 was either formed by dealkylation at an amine group in the cyclam ring or hydroxylation of a secondary nitrogen atom in the cyclam. Since the cyclam ring is involved in ligand binding to the active site of the receptor, it seems unlikely that the radioactive metabolite has significant affinity for CXCR4 (data not shown).

A potential bias in our study is that we did not assess the effect of the drug on tracer metabolism. Our previous study and other studies described in the literature, however, indicate that the drug Plerixafor® is not metabolized in vivo and is excreted unchanged in the urine [18]. We assumed therefore that Plerixafor® does not substantially compete with the metabolic route of the tracer and consequently, that pretreatment with Plerixafor® did not have any significant effect on the rate of metabolism of N-[^11^C]methyl-AMD3465. This assumption is supported by the fact that the plasma kinetics of the tracer were not affected by Plerixafor®.

Different compartmental models were used to fit the PET data, and the best model fit was obtained for 2TRCM. Pretreatment with the drug Plerixafor® resulted in a significant decrease in BP_ND_ and *V*_T_, indicating that specific tracer uptake could be inhibited by saturation of the CXCR4 receptor. Logan and Patlak graphical analysis of the TACs obtained from the tumor could be better fitted by Logan than Patlak analysis. This confirms that the binding of N-[^11^C]methyl-AMD3465 to the CXCR4 receptors is reversible. Furthermore, estimation of total blood volume (*V*_b_) was included in the analysis, since it can be affected by treatment with drugs [19]. In this study, however, we did not find any effect of Plerixafor® on tumor blood volume.

PET is a noninvasive imaging technique, which can provide insight into the relationship between receptor occupancy and drug efficacy, provided a suitable radioligand is available [20]. In the second part of our study, the feasibility of determining CXCR4 receptor occupancy by Plerixafor® using N-[^11^C]methyl-AMD3465 PET was assessed. For this purpose, both *V*_T_ and BP_ND_ values obtained from 2TRCM and Logan analysis were used to estimate the CXCR4 receptor occupancy. For occupancy measurements, a reliable fit with high reproducibility is necessary. Our data showed that *V*_T_ obtained from either 2TRCM analysis or from Logan analysis were highly correlated and gave a similar estimation of receptor occupancy. In contrast, *V*_T_ showed only a moderate correlation with BP_ND_ and occupancy values obtained from the BP_ND_ could not be reliably fitted to calculate the ED_50_. Theoretically, BP could give more accurate results, because it only relies on the receptor binding parameters *k*_3_ and *k*_4_, whereas *V*_T_ can also be affected by tracer delivery and tissue clearance. In theory, one would therefore expect that BP_ND_ is the parameter of choice to calculate the receptor occupancy, since it is independent of physiological parameters that could be affected by drug pretreatment, like blood flow, perfusion, non-specific binding, etc. However, BP_ND_ relies on the accurate estimation of the *k*3 and *k*4 values with the 2TRCM. Since the 2TRCM requires simultaneous estimation of five parameters (*K*1, *k*2, *k*3, *k*4 and *V*_b_), curve fitting may sometimes be difficult, and the estimated values for *k*3 and/or *k*4 can be highly sensitive to noise, especially when the number of available receptors is low, for example, in case of high receptor occupancy. Logan graphical analysis was a more stable modeling approach which can be used even when the 2TRCM does not fit properly.

A potential limitation of this study is that the effect of the endogenous ligand CXCL12 was not assessed. However, the effect of changes in binding of CXCL12 to CXCR4 on occupancy calculations was probably negligible, because all animals likely had comparable CXCL12 levels, as they received the same treatment. Another limitation is that the C6 tumors express both CXCR4 and CXCR7 [21]. Both CXCL12 and Plerixafor® can bind to both CXCR4 and to CXCR7 [22]. Since N-[^11^C]methyl-AMD3465 may also have some affinity to CXCR7, both CXCR4 and CXCR7 may have contributed to the occupancy measured in this study. These issues need to be further addressed in future studies.

## Conclusion

We have demonstrated that N-[^11^C]methyl-AMD3465 can be used to quantify CXCR4 receptor occupancy in tumors with PET. Tracer kinetics can be easily quantified by compartment modeling using the 2TRCM, which gives comparable estimates of *V*_T_ as Logan graphical analysis. Estimation of the BP_ND_ from the 2TRCM appears to be sensitive to noise. N-[^11^C]methyl-AMD3465 PET seems to be a useful tool to establish the relationship between drug dose and CXCR4 receptor occupancy in vivo. This technique could easily be translated to applications in humans, like patient-tailored, individualized therapy monitoring, and development of new drugs for CXCR4 receptors.
